# Assessment of the Effect of Attention-Deficit Hyperactivity Disorder on Choroidal Thickness Using Spectral Domain Optical Coherence Tomography

**DOI:** 10.14744/bej.2021.09821

**Published:** 2021-09-17

**Authors:** Serkan Akkaya, Dondu Melek Ulusoy, Hatice Dogan, Mahmut Erkam Arslan

**Affiliations:** 1. Department of Ophthalmology, Ankara Training and Research Hospital, Ankara, Turkey; 2. Department of Ophthalmology, Kayseri Training and Research Hospital, Kayseri, Turkey; 3. Department of Child and Adolescent Psychiatry, Kayseri Training and Research Hospital, Kayseri, Turkey

**Keywords:** Attention deﬁcit, choroid, hyperactivity, optical coherence tomography

## Abstract

**Objectives:**

This study was a comparison of the thickness of the choroid in pediatric patients with attention-deficit hyperactivity disorder (ADHD) and healthy pediatric controls.

**Methods:**

This study was comparative, cross-sectional, and observational in design. The healthy controls were age- and sex-matched with the members of the ADHD group and had no history of psychosis. Choroidal thickness was determined using spectral-domain optical coherence tomography.

**Results:**

A total of 138 patients were enrolled with a male:female ratio of 54 (69.2%): 24 (30.8%) in the ADHD group and 41 (68.3%): 19 (31.7%) in the control group (p=0.910). The ADHD patients had a mean age of 9.4±1.9 years (range: 6-12 years) and the controls had a mean age of 9.9±2.2 years (range: 6–12 years) (p=0.213). The ADHD group (n=78 eyes tested) had a significantly higher mean choroidal thickness at 1.5 mm (temporal-to-fovea, TTF) measurement than the controls (n=60 eyes tested) (281.12±46.63 μm vs. 264.40±48.61 μm, p=0.042). There were no significant differences in any of the other choroidal thickness measurements (p>0.05).

**Conclusion:**

The choroidal thickness measurement (TTF) at 1.5 mm was significantly greater in the ADHD patients. These findings suggest that choroidal thickness alterations may have a potential role in the underlying etiology of ADHD.

## Introduction

Attention-deficit hyperactivity disorder (ADHD) is a prevalent pediatric neurodevelopmental condition (1) that presents with impulsivity, hyperactivity, as well as an increase in distractibility (2). While the etiology of ADHD is not fully understood, recent findings suggest that it is due to an interaction of environmental and genetic contributors (3).

 Despite phenotypic differences ADHD, autism spectrum disorder, and schizophrenia (SZ) share many components in the etiological and pathophysiological pathways (4). Evidence of genetic overlap also suggests that these neurodevelopmental disorders can be accepted as a continuum (5).

Microvascular abnormalities appear as a potential contributor to the pathophysiology of SZ as shown by genetic, neuroimaging, postmortem gene expression, and morphological studies (6). Retinal vascular abnormalities such as venular dilation were also shown in SZ (7).

Reports have shown that abnormal brain structure is linked to ADHD, and comprehensive decreases in total brain volume have been observed in children with this condition (8-10) similar to SZ (11). The optic nerve and retina are considered to be part of the central nervous system (CNS) (12). Studies demonstrated that retinal nerve ﬁber layer (RNFL), macular thickness, as well as the foveal avascular zone (FAZ) may be altered in ADHD patients (13-17). Based on the vascular theory of these neurodevelopmental disorders we hypothesized that changes may also occur in the choroidal vasculature in ADHD.

Optical coherence tomography (OCT) is an imaging modality that is noninvasive, and is able to capture cross-sectional scans of the retina with high resolution (18). Advances in OCT technology made the imaging of the deeper structures possible (e.g., choroid) (19-22). The goal of the current study was to determine effects of ADHD on choroidal thickness via spectral-domain–OCT (SD-OCT).

## Methods

### Patients

This non-interventional, comparative, and cross-sectional study included 78 newly diagnosed ADHD patients (6–12 years old) who were recruited from the Department of Child and Adolescent Psychiatry of the single tertiary referral hospital between August 2017 and July 2019. The hospital’s ethics committee gave its approval for this study, which was conducted following the tenets of the Helsinki Declaration. The parents or legal guardians of the included children provided informed consent for the study.

This study also included 60 healthy subjects as a control group (matched for age and sex to the ADHD group) who were enrolled from the Ophthalmology Department of the single tertiary referral hospital. These healthy controls were subjected to a routine eye examination and were determined to have no ophthalmologic disorders, psychiatric complications, and/or history of medication.

All subjects included in this study were assessed by a psychiatrist specializing in children and adolescents using the Schedule for Affective Disorders and SZ for School-Age Children-Present and Lifetime Version-Turkish Version (K-SADS-PL-T) (23, 24).

All included subjects underwent dilated funduscopic examinations. Inclusion criteria were as follows: having a best-corrected visual acuity (BCVA) ≥20/20, a refractive error (SE) of ±2 diopters, and an intraocular pressure of <21 mmHg. Exclusion criteria were as follows: not being able to complete the OCT examination, previous intraocular surgery, glaucoma, organic eye diseases, strabismus, cataract, laser treatment, any conditions of the retina, and having any systemic illness, immune disorder, or neurological disease.

### Procedures

Every included subject was given 1% cyclopentolate (3 drops, Cyclogyl; Alcon Couvreur, Belgium) every 5 min, and cycloplegic refraction was performed 45 min after the initial treatment. Each subject had 5 autorefractor readings within 0.25 D of the other via a Tonoref II autorefractor/tonometer (Nidek Co. Ltd.). The following formula was used to calculate SE: spherical sum + 1/2 cylindrical error.

All subjects underwent other clinical assessments, including BCVA, extraocular movements, slit lamp examination, intraocular pressure, average central keratometry, and central thickness of the cornea (Scheimpflug camera, Wetzlar, Germany). In addition, the IOL Master (Carl Zeiss Meditec, CA, USA) was used to measure the eye’s axial length.

### SD-OCT Measurements

A Heidelberg Spectralis SD-OCT imaging platform (Spectralis, Germany) and an EDI program were used to obtain images for measuring choroidal thickness. Poor quality scans (scores under 20) and unclear images were excluded from the analysis.

**Figure 1.**
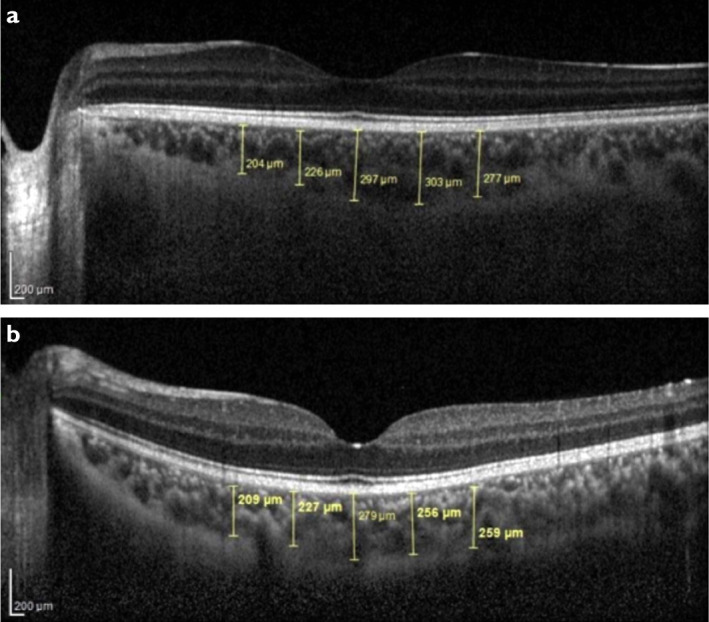
OCT images of macular choroidal thicknesses. (a) OCT scan of an ADHD patient (7-year-old). Yellow lines designate choroidal thickness measurements taken at the fovea, 0.75 mm and 1.5 mm TTF, and 0.75 mm and 1.5 mm nasal to the fovea. (b) OCT image of 8-year-old control subject.

As previously described, EDI-OCT was used to determine subfoveal choroidal thickness (19). Measurements were taken spanning the outside edge of the hyper-reflective choroid line to the innermost scleral edge. Macular choroidal thickness measurements were determined at the subfoveal region in 750-μm intervals between the fovea, 1.5 mm nasal, and temporal from the foveal center (Fig. 1). To evade any diurnal disparities, these measurements were taken between 12:00 and 2:00 PM.

### Statistical Analysis

All analyses were made in SPSS v 22.0 (SPSS, IL, USA). The data distribution was assessed with a Kolmogorov–Smirnov test. Parametric data were assessed via Student’s t-test and non-parametric data underwent analysis via Mann–Whitney U test. A Chi-square test was used to compare group gender distributions. Values of p&#x2264;0.05 were determined to be significant.

## Results

A total of one hundred thirty-eight subjects were enrolled in this study. The male/female ratio was 54 (69.2%)/24 (30.8%) for the ADHD group and 41 (68.3%)/19 (31.7%) for the control group. There was no difference in gender distribution between the controls and the ADHD patients (p=0.910). The average age of ADHD patients was 9.4±1.9 years (range (min:max), 6:12 years) and was 9.9±2.2 years (range, 6:12 years) in controls (p=0.213).

In addition, differences were not significant between the ADHD patients and controls with regards to SE, mean central keratometry, central corneal thickness, or axial length (Table 1).

**Table 1.** Analyses of spherica lequivalent, average central keratometry, central corneal thickness, and axial length

**Parameters**	**ADHD group (n=78)**		**Control group (n=60)**		**p**
	**Mean±SD**	**Median**	**Mean±SD**	**Median**	
Spherical equivalent (D)	0.00±0.60	–0.12	–0.08±0.88	0.12	0.67
	(–1.38–1.88)		(–1.88–1.63)		
Average central keratometry (D)	43.3±1.5	43.2	43.5±1.3	43.4	0.29
	(39.30–47.0)		(40.4–46.0)		
Central corneal thickness (μm)	547.2±28.9	541	545.0±28.7	542	0.65
	(470–620)		(487–616)		
Axial length (mm)	22.9±0.6	22.8	23.1±0.7	23.2	0.08
	(21.7–24.3)		(21.6–24.7)		

ADHD: Attention deﬁcit hyperactivity disorder; D: Diopters; SD: Standard deviation.

The ADHD patients had significantly higher 1.5 mm temporal to the foveal center (TTF) choroidal thickness than the controls (281.12±46.63 vs. 264.40±48.61 μm, respectively, P = 0.042). Although choroidal thickness values also in other locations in the ADHD group was higher than the control group’s values, the differences were not significant (Table 2).

**Table 2.** Mean choroidal thickness measurements at various locations in the attention-deficit hyperactivity disorder and control groups

**Location (mm)**	**Choroidal thickness (μm)**		**p**
	**ADHD group (n=78)**	**Control group (n=60)**
	**Mean±SD**	**Mean±SD**
Subfoveal	307.69±51.16	298.73±63.13	0.359
Temporal 0.75^a^	287.85±51.20	275.23±58.41	0.179
Temporal 1.5	281.12±46.63	264.40±48.61	0.042
Nasal 0.75	266.00±53.34	257.36±64.04	0.389
Nasal 1.5	235.80±49.03	233.88±64.90	0.843

^a^Denotes the position: 0.75 mm temporal to fovea. The same naming convention is used for subsequent entries. ADHD: Attention deﬁcit hyperactivity disorder; SD: Standard deviation

Age, SE, axial length, mean central keratometry, and central corneal thickness were not associated with choroidal thickness 1.5 mm TTF in ADHD patients (p=0.323, 0.977, 0.714, 0.053, and 0.228, respectively).

## Discussion

In this study, SD-OCT was used to assess choroidal thicknesses of patients with ADHD. The ADHD group had increased 1.5 mm TTF thickness of the choroid compared to controls. There were no correlations between the 1.5 mm TTF choroidal thickness measurements and demographic or ocular parameters.

OCT has previously been used to assess ADHD patients (13-15). Hergüner and colleagues examined macular thickness and volume as well as RNFL thickness in patients with ADHD. In that study, ADHD patients had a significantly reduced RNFL thickness in the nasal quadrant compared to controls. In addition, they did not observe differences in parafoveal macular thickness between groups, however, they did not analyze central macular thickness in their study (13). The recent study observed lower central macular thickness in patients with ADHD compared to controls (14). In a subsequent study, Bodur and colleagues compared ganglion cell layer (GCL), RNFL thickness, and optic nerve thickness in healthy controls and ADHD patients. That study also included the ADHD + oppositional defiant disorder (ODD) subjects. While RNFL thickness did not signifcantly differ among the two groups, the ADHD group had much lower optic nerve and GCL thickness than the healthy controls. Further, they concluded that the comorbidity of ODD had no additional effect on thinning (15).

To the best of our knowledge, the current study is the first to determine choroidal thickness in ADHD subjects with SD-OCT.

The choroid consists of connective tissue, melanocytes, blood vessels, ﬁbroblasts, and resident immunocompetent cells (25). Choroidal thickness is thought to be associated with the number of large vessels, their diameters, and amount of connective tissue in the choroid. It is thicker temporally than nasally. Although choroidal thickness was increased in all locations in ADHD group only the difference in 1.5 mm TTF location was significant. This may be explained by the denser vascular network temporally which may be affected more by the pathophysiologic processes. Results of the current study suggest that choroidal thickening at the 1.5 mm TTF region may be relevant to these factors in ADHD patients.

Grönlund et al. (26) reported that ADHD patients present with subtle optic nerve morphological changes and vasculature alterations in the retina. That study suggests an early developmental disturbance of the neural/vascular tissues in the CNS.

Results of the current study reveal an increased choroidal thickness at the 1.5 mm TTF region for the ADHD patients versus controls. It is unclear why the choroidal thickness increased at this region in ADHD patients. However, the variance may be caused by disturbances in vascular development.

Beyond the CNS, blood markers of pro-/anti-inflammatory states have been reported in ADHD (27). It has been hypothesized that these states may also contribute to increased choroidal thickness in ADHD.

A recent study by Tarakcioglu et al. (17) quantitatively compared the choroidal and retinal microvasculature using OCT angiography (OCTA) in patients with ADHD and found increased parafoveal thickness and choriocapillary ﬂow area in a methylphenidate treatment group compared to a no treatment control group.

Other parameters were not significantly different, including FAZ area, foveal and perifoveal density, FAZ perimeter, and thickness levels. However, that study did not compare OCTA parameters between ADHD patients and healthy controls.

It has also been hypothesized that blood turbulence, or varying orientations of flow, may cause variations in macular choroidal thickness in ADHD, as well as increasing the 1.5 mm TTF region choroidal thickness.

OCTA is a recently developed imaging technique that reveals changes in choroidal thickness and the vasculature. OCTA may provide evidence for the increased choroidal thickness in the patients with ADHD.

There are several limitations to our study. There wasn’t an independent examiner for the evaluation of the OCT scans and measurements were made manually. Measurements were taken on a single day. Making measurements on several occasions would have increased the reliability of the data. Considering the age of our study group this was not appropriately feasible. We weren’t able to evaluate the anxiety caused by the eye examination. This might also have caused an increase in choroidal thickness. The results of our study don’t allow us to establish a casual relationship between increased choroidal thickness 1.5 mm TTF and ADHD. Further studies are needed to support that choroidal thickness could be used as a marker for the ADHD.

## Conclusion

Results of the current study show increased choroidal thickness at the 1.5 mm TTF region in ADHD patients. Choroidal thickness should be considered and further investigated in a larger population and using newer imaging modalities like OCTA, so that its role in the underlying etiology of ADHD can be fully elucidated.

## Disclosures

### Ethics Committee Approval:

The present study was approved by the local ethics committee and informed consent was obtained from all participants or their parents or legal representatives. This study was conducted in accordance with the Declaration of Helsinki.

### Peer-review:

Externally peer-reviewed.

### Conflict of Interest:

None declared.

### Authorship Contributions:

Involved in design and conduct of the study (SA, DMU, HD, MEA); preparation and review of the study (SA, DMU, HD, MEA); data collection (SA, DMU, HD, MEA); and statistical analysis (SA).
